# Community-oriented primary care: The missing link

**DOI:** 10.4102/phcfm.v2i1.260

**Published:** 2010-12-07

**Authors:** Steve Reid

**Affiliations:** 1 Primary Health Care Directorate, Faculty of Health Sciences, University of Cape Town.

**Keywords:** community involvement, primary care, population health, missing link

## INTRODUCTION

We pay lip-service to community involvement and to looking after a ‘population-at-risk’. Understanding our communities properly is the missing component of our practice and our teaching between the patients who present themselves for care and the communities that we seek to serve. Conceptually, community-oriented primary care (COPC) is the connection between the concepts of Family Medicine and Primary Health Care. Or maybe it could be seen as the glue that keeps them together. In the African context of much fewer health professionals per population than in the northern hemisphere, it becomes essential if we are to do anything more than just cope with emergencies and the end effects of entrenched disease:

Every apparent success must be measured against the needs of all. Every effort, every cluster of resources must be divided by the total number of people. The insistence of using this denominator – all the people – has profound social, political, ethical and educational implications.^1^

As clinicians we concentrate almost exclusively on personal care of the individual patient which is like the tip of an iceberg of ill-health or potential illness. But we do not give enough attention to those who do not or cannot present themselves as ill, the so-called population-at-risk, which is the rest of the iceberg that we usually cannot see. We need to focus on access to healthcare for those who do not come for care – those who are too old, or too young, or too poor, or too scared, or live too far away, to get the help that they need.

## COMMUNITY-ORIENTED PRIMARY CARE METHODS

COPC gives us the tools to combine preventive, promotive and curative approaches by focusing our efforts on a defined community and prioritising the issues.^2^

### How do we go about reaching those who do not come?

We need to start with our existing practice population, those whom we already know and who do come. And then we need to extend that care to family, relatives, friends and contacts, including efforts to prevent illness and promote health. These activities typically include home visits, contact tracing, case-finding, screening and health education. This can be further extended into the community through patient interest groups and local non-government organisations. At a broader level such activities can be taken to scale through formal structures, that is tribal, local government and municipal structures.

Access to health care is the crucial issue and one that we have to give attention to in addition to looking after those who do come for care. It is not just a token of altruism, this is part of our professional duty as family physicians. This is not public health; it is COPC – the planned integration of primary care with public health in a defined community. Linking activities (like home visits) that connect the individual to the community perspective are crucial. The key starting point is a defined community (i.e. knowing your denominators and defining the boundaries) because without a defined community you cannot plan health for all and you end up planning for the health of a select few.

Secondly, a process of prioritisation needs to be carried out because communities are complex and dynamic and have multiple problems and crises simultaneously. This means that you have to possess the evidence to back up your priorities. We need to focus on health and not illness, that is, keeping people healthy and not just treating disease

A team approach is necessary for COPC, because one person cannot provide care to communities of more than 1000 people. Outside of the hospital or the medical setting, the doctor is not necessarily the one who is in charge.

This conference represents a crossroads for Family Medicine in Africa; I see two possible directions from here into the future:

- The one direction continues with the ‘northern’ model of personal medical care that deals only with those who present themselves for care and has the danger of becoming elitist, because a single doctor can only provide direct care to a limited number of people. We face the danger that Family Medicine becomes only for those who can afford private practice and nurses or clinical assistants providing primary care to the rest.
- The other direction takes on the population-based approach to a defined community and systematically addresses the barriers to access and the quality of care for everyone within that boundary, using the whole primary health care team. The five-star doctor is the leader of the clinical team, but delegates different types of care to different members of the team and works with lay members in a contributory way.

### The five-star doctor

Of the five attributes of the five-star doctor, which is the predominant one? Which one defines a doctor’s identity? By tacit agreement, the consensus has always been that the family practitioner is first and foremost a clinician and the other four roles are additional responsibilities:

- care provider and clinician
- health manager
- team leader and decision maker
- teacher and communicator
- community leader

But what if we redefined the role for the African context, to say that this person needs to be first and foremost a *teacher*, rather than a clinician? With a team of around 100 health personnel of different categories to provide comprehensive care to a population of 100 000 people, there is a great need for basic training and ongoing professional development. Our graduates therefore need to gain the very specific skills surrounding the act of teaching; they must be capable of passing on to their respective teams everything that we teach them.

Or if we say that we primarily need *leadership* and to develop individuals with initiative and vision? This attribute could even define the Family Practitioner in Africa. If the primary role of the generalist doctor in an African context is as leader of the primary health care team, then we need an educational process that produces leaders and not followers. We need graduates with initiative and vision who are able to inspire the primary health care teams that they work in. But if the primary role is that of community leader, this is a tall order and probably not a feasible one.

### Training

The point is that if we want a five-star doctor for Africa, we need to start training them; this means that we need to take seriously the process of development and assessment of each of the five components and not just the clinical component. Our standard method of assessment focuses almost exclusively on the family physician as care provider to the individual patient and the other dimensions are marginalized. We assume that if doctors are good clinicians, all the other things will happen as well. Unfortunately, they do not. Doctors are not necessarily good community leaders or good teachers – they have to learn how to lead well and have to be taught how to teach. This is our work as educators.

The second question can be posed as to how we are supposed to teach it, if it is so poorly practiced. How do we assess and examine these skills and this approach? Does a practitioner who is more community-oriented conduct consultations differently to someone who does not know their community? What could we see in their practice that is different?

I believe that the following attributes and practices of a family physician who is truly community oriented, can be measured and replicated.

### The community-oriented practitioner:

sees each consultation as the ‘tip of the iceberg’ of other similar problems in the community thereby representing a ‘population at risk’
questions each patient about the generalisability of the presenting problem, for example, the social determinants, the predisposing factors, infectivity and/or contacts
devises a management plan not only for the presenting patient, but also for those who are directly affected by the same conditions that lead to the presentation, for example, other family members, contacts and/or groups of people who may be at risk
knows the ‘big picture’ in terms of the population that he or she serves – its demographics, strengths and weaknesses and its unique characteristics
is consciously aware of the highest priorities for the health of that population, not just the disease profile
works in a team with other practitioners on health systems to promote access to care for vulnerable or marginalised groups in the community
is aware of and contributes a medical perspective to agencies involved in addressing the major problems affecting health of a defined community on a preventive and promotive basis

## CONCLUSION

As Family Physicians in Africa, we will fail in our duty to our communities if we continue with the medical model of health care that caters only to those who present themselves for care. By combining personal care with the broader PHC approach, using the tools of community-oriented primary care, we will build a much more robust and appropriate model of health care for our situation.
